# Correlation of *Salmonella enterica* and *Listeria monocytogenes* in Irrigation Water to Environmental Factors, Fecal Indicators, and Bacterial Communities

**DOI:** 10.3389/fmicb.2020.557289

**Published:** 2021-01-08

**Authors:** Ganyu Gu, Laura K. Strawn, Andrea R. Ottesen, Padmini Ramachandran, Elizabeth A. Reed, Jie Zheng, Renee R. Boyer, Steven L. Rideout

**Affiliations:** ^1^Eastern Shore Agricultural Research and Extension Center, Virginia Tech, Painter, VA, United States; ^2^Center for Veterinary Medicine, US Food and Drug Administration, Laurel, MD, United States; ^3^Center for Food Safety and Applied Nutrition, US Food and Drug Administration, College Park, MD, United States; ^4^Department of Food Science and Technology, Virginia Tech, Blacksburg, VA, United States

**Keywords:** bacterial communities, ABIOTIC factors, irrigation water, Listeria monocytogenes, Salmonella

## Abstract

Outbreaks of foodborne illnesses linked to fresh fruits and vegetables have been key drivers behind a wide breadth of research aiming to fill data gaps in our understanding of the total ecology of agricultural water sources such as ponds and wells and the relationship of this ecology to foodborne pathogens such as *Salmonella enterica* and *Listeria monocytogenes*. Both *S. enterica* and *L. monocytogenes* can persist in irrigation water and have been linked to produce contamination events. Data describing the abundance of these organisms in specific agricultural water sources are valuable to guide water treatment measures. Here, we profiled the culture independent water microbiota of four farm ponds and wells correlated with microbiological recovery of *S. enterica* (prevalence: pond, 19.4%; well, 3.3%), *L. monocytogenes* (pond, 27.1%; well, 4.2%) and fecal indicator testing. Correlation between abiotic factors, including water parameters (temperature, pH, conductivity, dissolved oxygen percentage, oxidation reduction potential, and turbidity) and weather (temperature and rainfall), and foodborne pathogens were also evaluated. Although abiotic factors did not correlate with recovery of *S. enterica* or *L. monocytogenes* (*p* > 0.05), fecal indicators were positively correlated with incidence of *S. enterica* in well water. Bacterial taxa such as *Sphingomonadaceae* and *Hymenobacter* were positively correlated with the prevalence and population of *S. enterica*, and recovery of *L. monocytogenes* was positively correlated with the abundance of *Rhizobacter* and *Comamonadaceae* (*p* < 0.03). These data will support evolving mitigation strategies to reduce the risk of produce contamination by foodborne pathogens through irrigation.

## Introduction

Fresh produce is increasingly recognized as a common vehicle for transmission of foodborne pathogens (Brandl, 2006; Franz and van Bruggen, 2008; Nuesch-Inderbinen and Stephan, 2016; Carstens et al., 2019). Ranked in the top five pathogens contributing to illnesses, hospitalizations, or deaths in the United States during outbreaks of foodborne diseases, *Salmonella enterica* spp. and *Listeria monocytogenes* are considered to be common foodborne pathogens associated with fresh produce (CDC, 2018). Consumption of *S. enterica* contaminated produce has led to several multistate and international outbreaks in recent years (CDC, 2005, 2007; Greene et al., 2008; Bennett et al., 2015). *L. monocytogenes* was responsible for a 2011 produce-borne outbreak in the United States, with 147 illnesses, 33 deaths, and 1 miscarriage, due to consumption of cantaloupe (CDC, 2012). In addition, a considerable number of produce recalls have occurred in the past years as a result of contamination by the two pathogens (FDA, 2007-2017).

Irrigation water may play an important role in contaminating vegetables and fruits with foodborne pathogens (Solomon et al., 2002; Islam et al., 2004; Hintz et al., 2010; Oliveira et al., 2011; Weller et al., 2015). Agricultural water can be contaminated *via* sewage overflows, polluted storm water runoff, and agricultural runoff. The 2005 multistate *S. enterica* Newport outbreak associated with tomato was reported to be related to contaminated irrigation water (CDC, 2007). After irrigation with contaminated water, the bacteria can adhere to plants, enter into plants, and translocate within infested plants. These pathogens would be difficult to be completely removed during typical washing and disinfection procedures and may persist and multiply at any point along with the farm-to-fork continuum from production to consumption (Solomon et al., 2002; Franz and van Bruggen, 2008; Miles et al., 2009; Barak et al., 2011; Gu et al., 2011, 2013c; Zheng et al., 2013). Therefore, minimizing the risk of contamination by human bacterial pathogens during the pre-harvest period is essential to reducing foodborne illness risks.

Fecal pollution, including *S. enterica* spp. and *L. monocytogenes*, is traditionally evaluated with fecal indicator bacteria like fecal coliforms and generic *Escherichia coli* (EPA, 2002). Contradictory results have been reported as to the correlation between indicator organisms and the occurrence of human pathogens in surface water (Burton et al., 1987; Rhodes and Kator, 1988; Chandran and Hatha, 2005; Ahmed et al., 2009; Chandran et al., 2011; Benjamin et al., 2013; Gu et al., 2013a; McEgan et al., 2013). Chigor et al. (2010) reported that some environmental factors, including water turbidity and concentrations of nitrate, phosphate, and chloride, were positively correlated with the population of fecal coliforms in a river used for fresh produce irrigation in Nigeria. A former study evaluating irrigation ponds in Georgia showed positive correlation between temperature, rainfall, populations of fecal coliform, and culturable bacteria and the occurrence of *E. coli* O157, and a negative relationship between the total nitrogen concentration, oxidation reduction potential (ORP), and dissolved oxygen concentration and the occurrence of this pathogen (*p* < 0.05; Gu et al., 2013a). Another survey conducted in central Florida presented different results on the relationship of environmental factors to the prevalence of *S. enterica* in surface water (McEgan et al., 2013). These studies showed regional differences for the prevalence of detected foodborne pathogens and the efficacy of indicators to predict the prevalence of the pathogens (Ahmed et al., 2009; Benjamin et al., 2013; Gu et al., 2013a; McEgan et al., 2013). It is necessary to evaluate and validate the efficacy of biological index organisms and physicochemical indicators on the prediction of foodborne pathogen contamination in major agricultural regions in the United States. Furthermore, previous studies about the impacts of bacterial communities on the prevalence of foodborne pathogens in irrigation ponds showed the probability of identifying alternative indicator microorganisms to economically and reliably predict the presence or absence of foodborne pathogens in irrigation water and the on-farm agricultural environment (Gu et al., 2013a,b).

In this study, the correlation between foodborne pathogens, *S. enterica* and *L. monocytogenes*, and contextual factors, including water parameters, weather information, fecal indicators, and bacterial community, has been analyzed to identify suitable indicators and potential suppressors of foodborne pathogens in irrigation water.

## Materials and Methods

### Water Sample Collection

**Figure 1:**
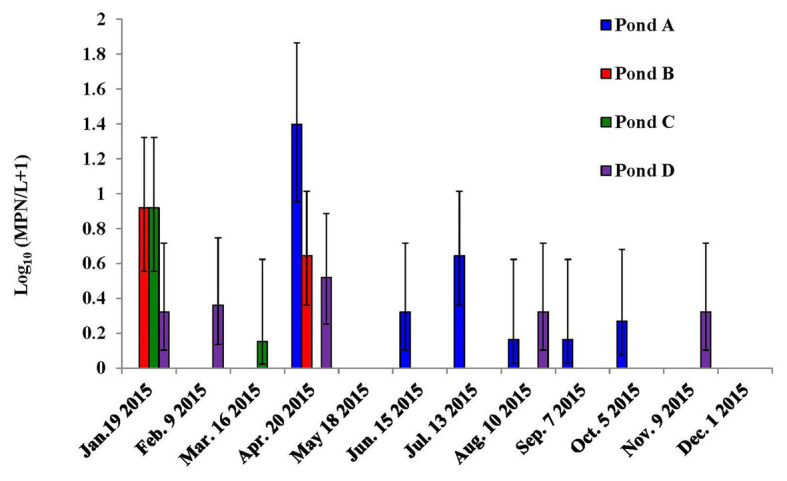
Dynamics of *Listeria monocytogenes* in the irrigation ponds of farms A–D on the Eastern Shore of Virginia (ESV). Bars represent 95% confidence intervals.

The study area is the Eastern Shore of Virginia (ESV), an important agricultural region in the United States including the top tomato production county in Virginia. From January to December 2015, 4 L of pond and well irrigation water samples were collected weekly from four vegetable farms (Farms A–D) on ESV for *S. enterica* spp. detection (Gu et al., 2019a). At one of the sampling times per month for *S. enterica* detection, 4-L water samples were also collected concurrently from the same irrigation ponds and wells for *L. monocytogenes* detection. Collected water samples were stored on ice in the field and transported to lab for further experiments. In total, 392 weekly pond and well irrigation water samples (2 water types × 4 farms × 49 weeks) were tested for *S. enterica*, and 96 monthly pond and well water samples (2 water types × 4 farms × 12 months) were collected for *L. monocytogenes* detection in this study (Figure 1).

### Water Parameter Measurements, Weather Information Collection, and Fecal Indicator Detection

During sampling, water samples were tested for temperature (°C), pH, pHmV, conductivity (S), dissolved oxygen percentage (%), dissolved oxygen charge, and oxidation reduction potential (ORP, mV) with an YSI® 6600 Multiparameter Sonde (Yellow Springs, OH) in the field. Water turbidity (nephelometric turbidity units, NTU) was tested using a HI98713 turbidity meter (Hanna instruments, Woonsocket, RI). MPN values (MPN/100 ml) of generic *E. coli* and coliforms were assayed using Colilert® (IDEXX Laboratories, Inc., Westbrook, ME) following the manufacturer’s instructions. A HOBO Micro Station (Onset Computer Corporation, Bourne, MA) was set up at each tested farm. Temperature (°C) and rainfall data (mm) 1 week prior to each sampling were collected for further analysis.

### *Salmonella enterica* and *Listeria monocytogenes* Detection and Most Probable Number Analysis

A most probable number (MPN) method was used to assess *S. enterica* concentration in collected water samples as described previously (Luo et al., 2014; Gu et al., 2019a,b).

*L. monocytogenes* was quantified by the MPN method mentioned above with some modifications. In brief, water samples (500, 100, and 10 ml) were added to equal volumes of sterile buffered *Listeria* enrichment broth (BLEB, Becton, Dickinson and Company, Sparks, MD) at 2x concentration in quadruplicate and incubated at 30°C for 24 h. Fifty microliters of each enrichment were streaked onto *Listeria monocytogenes* plating medium (LMPM, R&F Laboratories 0550M, Downers Grove, IL) for 48 h at 35°C. Positive colonies (turquoise convex) were confirmed by a cross-streaking method using modified Oxford agar (MOX; Becton, Dickinson) and by PCR amplification of *hlyA* gene (Furrer et al., 1991). Up to four positive colonies per plate were stored in 20% glycerol in a −80°C freezer.

The experiments of this study were performed by following the biosafety standard operating protocols approved by the Institutional Biosafety Committee at Virginia Tech (Permit No.: IBC # 17–051).

### Antimicrobial Susceptibility Test

Ninety-six well Sensititre™ Gram Negative NARMS Plates (Thermo Fisher Scientific, Waltham, MA) were used for antimicrobial susceptibility tests of selected *S. enterica* isolates according to the manufacturer’s instructions. Tested antibiotics included cefoxitin, azithromycin, chloramphenicol, tetracycline, ceftriaxone, amoxicillin/clavulanic acid 2:1 ratio, ciprofloxacin, gentamicin, nalidixic acid, ceftiofur, sulfisoxazole, trimethoprim/sulfamethoxazole, ampicillin, streptomycin, and streptomycin. Results were interpreted and antibiotic-resistant strains were defined according to the National Committee for Clinical and Laboratory Standards criteria (National Committee for Clinical Laboratory Standards, 2010).

### 16S rDNA High-Throughput Sequencing and Sequence Analysis

Water samples collected for both *S. enterica* and *L. monocytogenes* detection were selected for DNA extraction and subsequent 16S rDNA high-throughput sequencing analysis (4 farms × 2 water types (pond and well) × 12 months = 96). Two-hundred milliliters of each representative water sample was vacuum filtered through a 0.22 μm sterile nitrocellulose membrane (Millipore Corporation, Billerica, MA). Genomic DNA of bacteria captured by the membranes was extracted using a PowerWater® DNA Isolation Kit (MOBIO Laboratories, Inc., Carlsbad, CA) and stored at −80°C until use. Diversity of bacterial community, as well as abundance of bacterial species in water samples, was analyzed by 16S PCR amplicon sequencing using a Miseq Next Generation Sequencer (Illumina). Raw 16S rDNA amplicon sequences were trimmed for quality using Trimmomatic v0.32 and high-quality read pairs were merged using the FLASH tool. High-quality R1 reads that did not merge (due to a low quality R2 pair) were also included in downstream analysis. Final trimmed sequences were required to be at least 200 bp in length. During preprocessing, sequences were screened for PhiX contamination as well as chloroplast sequences using the RDP classifier trained on the GreenGenes 16S database.

Passing high-quality 16S rDNA sequences were analyzed using the Resphera Discovery protocol (RDP, Baltimore, MD). Briefly, sequences were clustered into operational taxonomic units using UCLUST (*de novo*) with a 97% identity threshold (Edgar, 2010). Representative members of each operational taxonomic unit (OTU) were assigned a consensus taxonomic lineage using the RDP classifier trained on the Resphera Discovery 16S database (minimum confidence 80%). Prior to downstream comparative analysis, samples were rarefied to 10,000 sequences per sample. Taxonomic abundance profiles and assignments were also classified using Kraken 2 classification tool (Wood and Salzberg, 2014) and Bracken abundance estimator (Lu et al., 2017), with the SILVA database (Quast et al., 2013).

### Statistical Analysis

With MPN analysis, a value of zero was given to any samples under the lower limit of detection. Upper limit values were given to any samples over the upper detection limit. *Salmonella enterica* and *Listeria monocytogenes* MPN values in pond and well irrigation water were log transformed using the formula log_10_(MPN + 1) to present the dynamics of population density for normalization. The log-transformed values were used for the following statistical analyses. Biserial correlation coefficients were calculated to evaluate the correlations between the environmental factors (water and weather parameters) and the occurrence of *S. enterica* (*n* = 396) or *L. monocytogenes* (*n* = 96) isolated from irrigation water (pond and well), and Pearson’s correlation coefficients were calculated to evaluate the correlations between the environmental factors and *S. enterica* or *L. monocytogenes* population density (Portney and Watkins, 2009). To analyze the correlation between foodborne pathogens and bacterial community in irrigation water samples, monthly *S. enterica* contamination prevalence and population density were chosen based on the selected sampling points used for both foodborne pathogen testing and sequencing (*n* = 96). Alpha diversity was evaluated for each sample using the Chao1 estimator (Chao, 1984) and Faith’s phylogenetic diversity (PD; Faith, 1992). Beta-diversity analysis was performed by Principal coordinates analysis (PCoA). The Mann-Whitney test was used for differential abundance analysis.

Statistical analysis was performed using SAS (SAS release 9.3, SAS Institute Inc., Cary, North Carolina). Except when stated otherwise, *p* < 0.05 were considered as statistically significant.

## Results

### Dynamics and Antimicrobial Resistance of *Salmonella enterica* in Irrigation Pond and Well Water

In our previous study, the prevalence and population density of *S. enterica* in irrigation tested ponds and wells were reported (Gu et al., 2019a).

In this study, all the *S. enterica* isolates collected from irrigation ponds in the previous study (Gu et al., 2019a) were further subjected to antimicrobial susceptibility test. Among the 270 *S. enterica* isolates, 12 isolates were identified to be resistant to at least one of the tested antibiotics, including four Thompson isolates resistant to ceftiofur or tetracycline, three Typhimurium isolates resistant to streptomycin, tetracycline, or amoxicillin/clavulanic acid 2:1 ratio, two Saintpaul isolates resistant to ceftriaxone or ciprofloxacin, two Newport isolates resistant to ceftriaxone or cefoxitin and amoxicillin/clavulanic acid 2:1 ratio, and one Larochelle isolate resistant to cefoxitin. All the identified well water isolates (*n* = 24) were sensitive to tested antibiotics.

### *Listeria monocytogenes* Prevalence and Population Density in Irrigation Pond and Well Water

Differences in *L. monocytogenes* occurrence were observed among ponds (Figure 1). The prevalence of *L. monocytogenes* in the four tested ponds of farms A, B, C, and D were 16.7, 25, 33.3, and 33.3%, respectively. The average MPN values of *L. monocytogenes* in the four ponds during the study were 0.93, 0.22, 1.66, and 6.44 MPN/L, respectively. For well water samples, *L. monocytogenes* was only isolated from Farm D in April (24 MPN/L, 95% confidence interval: 8 MPN/L – 72 MPN/L) and October (0.46 MPN/L, 0.07 MPN/L – 3.2 MPN/L).

### Correlation Between Foodborne Pathogens and Environmental Factors in Irrigation Water

There was no significant correlation between most water parameters and *S. enterica* occurrence in the tested ponds and wells (Tables 1 and 2; Figure 1 and Supplementary Figure S1), but *S. enterica* population in pond irrigation water was positively correlated with water pH (*p* = 0.038, Table 1). However, the significant correlation was mainly contributed by pond D (*p* < 0.01). Water pH was not significantly correlated with *S. enterica* MPN values in ponds A–C (*p* > 0.05). No significant correlations were identified between tested weather parameters (mean temperature and total rainfall) and *S. enterica* prevalence and levels in this study.

**Table 1:** Correlation between environmental factors and *Salmonella enterica* spp. in irrigation ponds on the Eastern Shore of Virginia (*n* = 196).

Contextual factors	Pearson’s correlation coefficients to *Salmonella enterica* population density [log(MPN +1)]	*p*^x^	Biserial correlation coefficients to *Salmonella enterica* prevalence	*p*^y^
Tm^a^	−0.007609538	0.91581	−0.034733071	0.62921
Conductivity^b^	−0.05719309	0.42665	−0.070175698	0.32891
DO percentage^c^	−0.04707085	0.51301	−0.066731859	0.35296
DO charge^d^	0.02336483	0.74519	0.00950965	0.89486
pH^e^	−0.148123873	**0.03831**	−0.13314992	0.06292
pHmV^f^	0.057121028	0.42649	0.005718715	0.93669
ORP^g^	−0.004021127	0.95563	−0.010955773	0.87948
Turbidity^h^	0.05239285	0.46584	−0.055239594	0.44222
Mean Tm^i^	−0.005098195	0.94455	−0.054947192	0.44471
Total Rf^j^	−0.055691718	0.43892	−0.119869831	0.09443
Coliform^k^	0.007890593	0.91261	0.007657521	0.91525
Generic *E. coli*^l^	0.005742038	0.93636	0.019552346	0.78564

Factors significantly correlated to *Listeria monocytogenes* population density or prevalence are in bold.

a Temperature (°C) of the pond water during sampling.

b Conductivity of water samples (S).

c Dissolved oxygen percentage (%).

d Dissolved oxygen charge.

e pH value of water samples [measured in standard units (S.U.)].

f pH value of water samples pH measured in millivolts (mV).

g Oxidation-reduction potential (mV).

h Turbidity of water samples (NTU, nephelometric turbidity units).

i Mean temperature (°C) in the pond region in the last week before sampling.

j Total rainfall (mm) in the pond region in the last week before sampling.

k Population of coliforms (CFU/100 ml).

l Population of generic *E. coli* (CFU/100 ml).

x Value of *p* of Pearson’s correlation coefficients.

y Value of *p* value of biserial correlation coefficients.

**Table 2:** Correlation between environmental factors and *Salmonella enterica* spp. in irrigation wells on the Eastern Shore of Virginia (*n* = 196).

Contextual factors	Pearson’s correlation coefficients to *Salmonella enterica* population density [log(MPN +1)]	*p*^x^	Biserial correlation coefficients to *Salmonella enterica* prevalence	*p*^y^
Tm^a^	−0.01691986	0.81413	−0.016205128	0.82169
Conductivity^b^	0.029190047	0.68465	0.037865224	0.59831
DO percentage^c^	−0.007406088	0.91801	−0.023832794	0.74056
DO charge^d^	−0.022610532	0.75321	−0.003246387	0.96449
pH^e^	−0.046222454	0.52022	−0.001031583	0.98891
pHmV^f^	0.029323838	0.68332	−0.007251536	0.92022
ORP^g^	0.058536029	0.41514	0.021279195	0.76729
Turbidity^h^	−0.010322127	0.88607	−0.032656483	0.65012
Mean Tm^i^	−0.020535885	0.77549	0.029568995	0.68087
Total Rf^j^	0.107148143	0.13501	0.036488403	0.61172
Coliform^k^	0.176633637	**0.01327**	0.205555488	**0.00385**
Generic *E. coli*^l^	0.210690813	**0.00304**	0.137615375	0.05443

Factors significantly correlated to *Listeria monocytogenes* population density or prevalence are in bold.

a Temperature (°C) of the pond water during sampling.

b Conductivity of water samples (S).

c Dissolved oxygen percentage (%).

d Dissolved oxygen charge.

e pH value of water samples [measured in standard units (S.U.)].

f pH value of water samples pH measured in millivolts (mV).

g Oxidation-reduction potential (mV).

h Turbidity of water samples (NTU, nephelometric turbidity units).

i Mean temperature (°C) in the pond region in the last week before sampling.

j Total rainfall (mm) in the pond region in the last week before sampling.

k Population of coliforms (CFU/100 ml).

l Population of generic *E. coli* (CFU/100 ml).

x Value of *p* value of Pearson’s correlation coefficients.

y Value of *p* value of biserial correlation coefficients.

**Figure 2:**
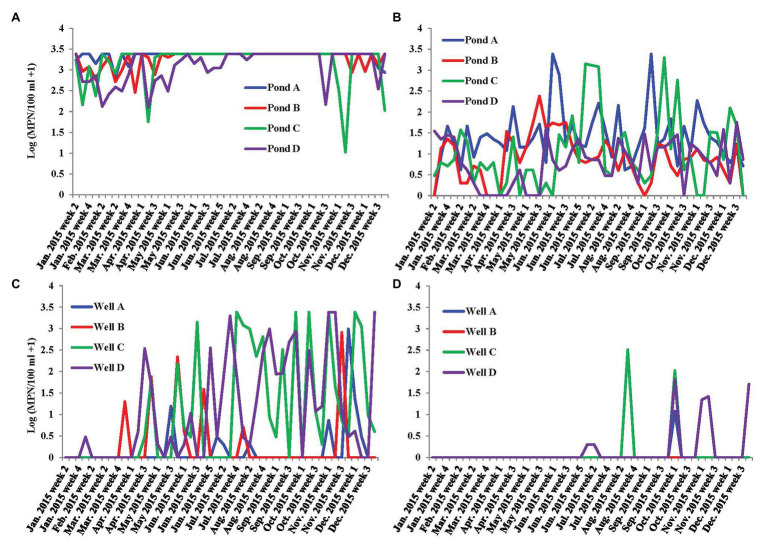
Dynamics of fecal indicators, coliform and generic *E. coli*, in the irrigation water of farms A–D on the ESV. **(A)** Most possible number (MPN) values of coliform in four tested irrigation ponds on ESV in 2015; **(B)** MPN values of generic *E. coli* in four tested irrigation ponds on ESV in 2015; and **(C)** MPN values of coliform in four tested irrigation wells on ESV in 2015; and **(D)** MPN values of generic *E. coli* in four tested irrigation wells on ESV in 2015.

Population of fecal indicators including coliforms and generic *E. coli* in irrigation pond and well water samples were examined during the study (Figures 2A–D). Population density of coliforms in most pond water samples exceeded the maximum detection limit (2419.6 MPN/100 ml; Figure 2A). There were no significant correlations between fecal indicators and the prevalence of *S. enterica* spp. in irrigation ponds (Table 1). However, coliform population was positively correlated with *S. enterica* prevalence and population densities in well water (Table 2). Similarly, the concentration of generic *E. coli* was also significantly positively correlated with *S. enterica* levels in well water (*p* < 0.01). The correlation between generic *E. coli* and *S. enterica* prevalence in wells is relatively significant (*p* = 0.05443). It is worthy to note that the significant correlation between fecal indicators and *S. enterica* was mainly due to the high correlation present in well water of farm D (*p* < 0.01).

The geometric means of generic *E. coli* in ponds A–D and wells A–D in the sampling period (in total of 49 samples in each water source) were 22.5, 6.3, 8.8, 5.7, 1.1, 1, 1.2, and 1.3 MPN/100 ml, respectively. The statistical threshold values of generic *E. coli* in ponds A–D and wells A–D in the sampling period (in total of 49 samples each) were 770, 62, 681, 28.7, 2.3, 0, 71.8, and 18.4 MPN/100 ml, respectively.

No environmental factors and fecal indicators measured in this study were significantly correlated to *L. monocytogenes* population and prevalence in irrigation ponds (Table 3). *L. monocytogenes* was only isolated from well D, which had a significant positive correlation with water turbidity (Table 4).

**Table 3:** Correlation between environmental factors and *Listeria monocytogenes* in irrigation ponds on ESV (*n* = 48).

Contextual factors	Pearson’s correlation coefficients to *Listeria monocytogenes* population density [log(MPN +1)]	*p*^x^	Biserial correlation coefficients to *Listeria monocytogenes* prevalence	*p*^y^
Tm^a^	−0.156916356	0.28688	−0.106512726	0.47124
Conductivity^b^	−0.006836878	0.96341	0.213382481	0.14534
DO percentage^c^	0.002466153	0.98676	0.078838887	0.59432
DO charge^d^	0.06095121	0.68069	0.218988495	0.13482
pH^e^	0.069182978	0.64034	0.105586622	0.47511
pHmV^f^	−0.111864227	0.44933	−0.176437183	0.23039
ORP^g^	0.164771709	0.26308	0.130927857	0.37511
Turbidity^h^	−0.110702399	0.45383	−0.118182688	0.42403
Mean Tm^i^	−0.218220547	0.13625	−0.16520481	0.26182
Total Rf^j^	−0.022657725	0.87882	−0.219150669	0.13461
Coliform^k^	−0.20828325	0.15559	−0.169018948	0.25085
Generic *E. coli*^l^	0.190283653	0.19518	0.254827787	0.08048
*Salmonella enterica* population density^z^	0.238520512	0.10255	−0.018594	0.90068

a Temperature (°C) of the pond water during sampling.

b Conductivity of water samples (S).

c Dissolved oxygen percentage (%).

d Dissolved oxygen charge.

e pH value of water samples [measured in standard units (S.U.)].

f pH value of water samples pH measured in millivolts (mV).

g Oxidation-reduction potential (mV).

h Turbidity of water samples (NTU, nephelometric turbidity units).

i Mean temperature (°C) in the pond region in the last week before sampling.

j Total rainfall (mm) in the pond region in the last week before sampling.

k Population of coliforms (CFU/100 ml).

l Population of generic *E. coli* (CFU/100 ml).

x Value of *p* value of Pearson’s correlation coefficients.

y Value of *p* value of biserial correlation coefficients.

z *Salmonella enterica* population density [log(MPN + 1)].

**Table 4:** Correlation between environmental factors and *Listeria monocytogenes* in irrigation well D on ESV (*n* = 12).

Contextual factors	Pearson’s correlation coefficients to *Listeria monocytogenes* population density [log(MPN +1)]	*p*^x^	Biserial correlation coefficients to *Listeria monocytogenes* prevalence	*p*^y^
Tm^a^	0.073992	0.81924	0.381534	0.22105
Conductivity^b^	0.113721	0.72491	−0.03904	0.90422
DO percentage^c^	0.034744	0.91464	0.039598	0.90278
DO charge^d^	−0.1599	0.61961	−0.03243	0.92038
pH^e^	0.166137	0.60584	0.081467	0.80129
pHmV^f^	−0.17156	0.59407	−0.03148	0.92283
ORP^g^	−0.45143	0.14075	−0.15318	0.63476
Turbidity^h^	−0.16751	0.60283	0.587402	0.04462
Mean Tm^i^	−0.22321	0.48561	0.095717	0.76732
Total Rf^j^	−0.11303	0.72659	−0.11945	0.71168
Coliform^k^	0.043451	0.89334	0.104528	0.74649
Generic *E. coli*^l^	−0.10588	0.74348	0.454206	0.13799

Factors significantly correlated to *Listeria monocytogenes* population density or prevalence are in bold.

a Temperature (°C) of the pond water during sampling.

b Conductivity of water samples (S).

c Dissolved oxygen percentage (%).

d Dissolved oxygen charge.

e pH value of water samples [measured in standard units (S.U.)].

f pH value of water samples pH measured in millivolts (mV).

g Oxidation-reduction potential (mV).

h Turbidity of water samples (NTU, nephelometric turbidity units).

i Mean temperature (°C) in the pond region in the last week before sampling.

j Total rainfall (mm) in the pond region in the last week before sampling.

k Population of coliforms (CFU/100 ml).

l Population of generic *E. coli* (CFU/100 ml).

x Value of *p* of Pearson’s correlation coefficients.

y Value of *p* value of biserial correlation coefficients.

### Bacterial Community in Irrigation Water and Relationship to Foodborne Pathogens

The 16S rDNA high throughput sequencing data have been submitted to NCBI with accession number PRJNA630202. Approximately 57,000 raw 16S rDNA gene sequences with read lengths above 200 bases were acquired for each tested water sample collected from irrigation ponds and wells on the ESV. These data were further culled using quality trimming methods described above to an average of 34,719 sequences per sample, with an average length of 269 bases for use in downstream analyses.

Alpha diversity (Simpson’s Reciprocal and Shannon index) of the bacterial community, represented by 16S rRNA gene amplicons using OTUs at 97% similarity, was significantly lower (Mann-Whitney test, *p* < 0.01) in well water samples compared to pond water samples. This trend continued regardless of whether the diversity was measured by observed OTUs (pond: 1415.18 ± 57.29 and well: 985.17 ± 54.09), phylogenetic diversity (whole tree, pond: 403.76 ± 13.75; well: 331.31 ± 4.54), and Chao1 (pond: 3640.51 ± 180.26 and well: 2004.24 ± 18.12). However, there was no significant difference regarding alpha diversity between water samples that tested positive or negative for *S. enterica* or *L. monocytogenes* (*p* > 0.05).

In general, the dominant bacterial phylum and classes in water samples from each water source and sampling time were not completely distinct from one another (Supplementary Figures S2–S5). The most abundant taxa (phylum) observed in water samples were members of Proteobacteria (51.98%), Bacteroidetes (12.89%), and Actinobacteria (9.38%).

**Figure 3:**
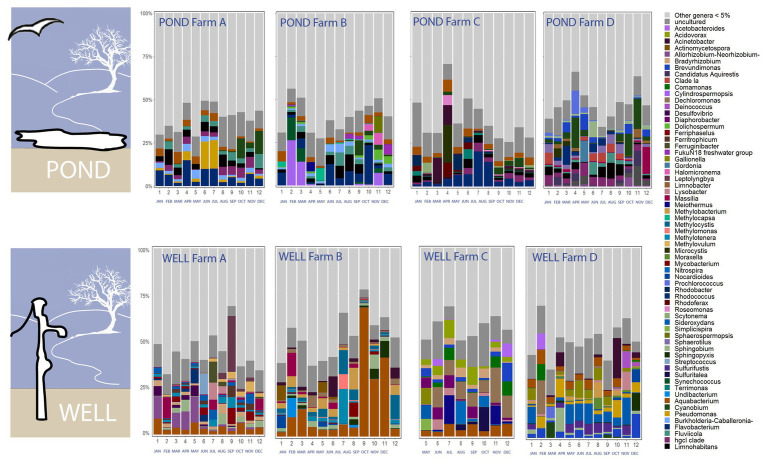
Dynamics of abundant bacterial genera (>5%) in irrigation ponds and wells during the 12 sampling months from four tested farms A–D on the ESV (sequencing data were not available for well water in farm C from January to April due to inadequate bacterial DNA extracted from water samples).

The relative abundance of phyla Proteobacteria, Firmicutes, Spirochaetes, and Acidobacteria, was significantly higher in well water, while the relative abundance of Bacteroidetes, Acinobacteria, Cyanobacteria, Verrucomicrobia, and Planctomycetes, was higher in pond water samples (Supplementary Table S1). The higher level of proteobacteria in well water was contributed by higher abundance of classes beta-proteobacteria, gamma-proteobacteria, and delta-proteobacteria (Supplementary Table S2). The abundance of class Sphingobacteria and Flavobacteria (under phylum Bacteroidetes), and Actinobacteria (phylum Actinobacteria) was higher in pond water (Supplementary Table S2). The relative abundance of class Spirochetes and Deinococci increased from August to December in samples from both pond and well water (Supplementary Figure S5). The dynamics of abundant bacterial genera (>5%) in irrigation water showed obvious shift during sampling months and disparities among farms in the tested agricultural region (Figure 3). The top three genera in pond water were *Flavobacterium* (average relative abundance: 5.25%), *Limnohabitans* (3.35%), and hgcI clade (*Actinobacteria*, 2.98%) and *Aquabacterium* (7.43%), *Dechloromonas* (2.73%), and *Sideroxydans* (2.61%) in well water samples.

**Figure 4:**
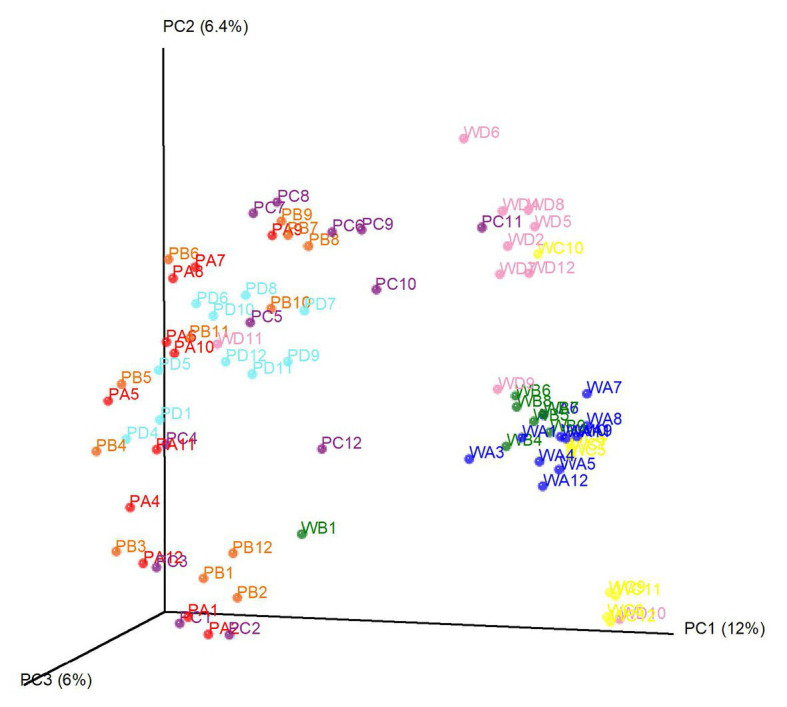
Principal coordinate analysis of the bacterial community in tested irrigation water samples. Each dot represents one water sample. Samples with the same color were collected from the same location. P denotes pond water and W denotes well water. Numbers behind denote sampling time (month).

Beta-diversity analysis was conducted based on Bray-Curtis distances and points were colored to present dominant factors associated with community composition (Figure 4). Based on the beta diversity analysis, we observed an effect of both water source and location. Water samples were clearly differentiated by source (pond or well). Certain well water samples were grouped by farm location, and some pond water samples were associated with sampling time. For example, among well samples, well D had a distinct community, while well A and well B were much closer in community composition. There was substantial overlap in composition among pond samples from all four locations. Nevertheless, samples that were positive and negative for foodborne pathogens (*S. enterica* and *L. monocytogenes*) could not be differentiated by the principle coordinate analysis.

**Figure 5:**
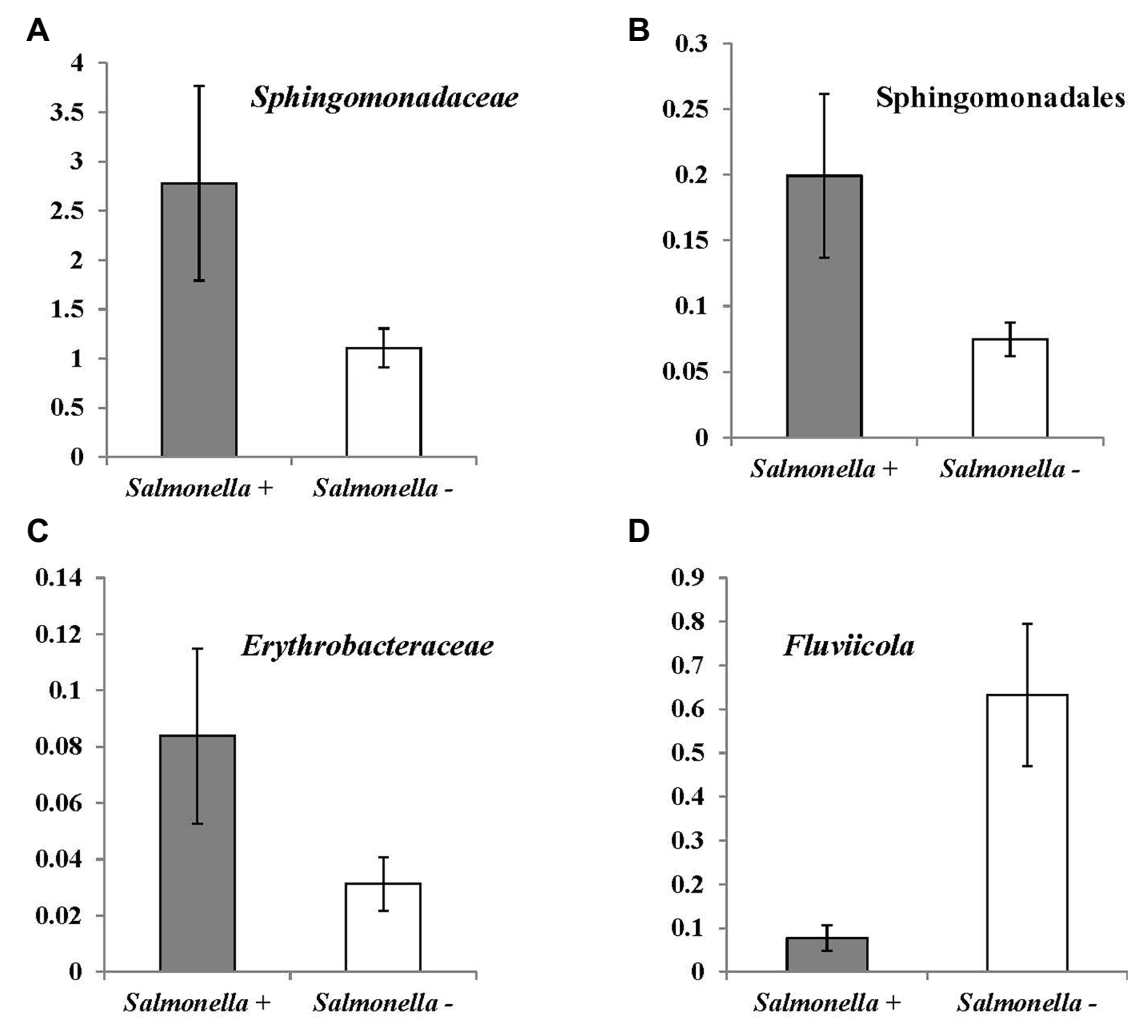
OTUs (at bacterial genus level) significantly correlated with *Salmonella enterica* prevalence in irrigation ponds by Mann-Whitney U test. **(A)** unidentified genus of family *Sphingomonadaceae* (*p* = 0.021); **(B)** unidentified genus of order Sphingomonadales (<0.007); **(C)** unidentified genus of family *Erythrobacteraceae* (0.038); and **(D)** *Fluviicola* (0.008).

**Figure 6:**
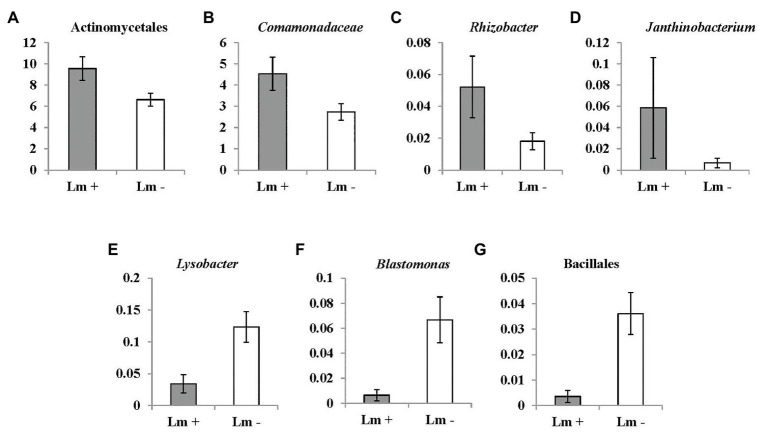
OTUs (at bacterial genus level) significantly correlated with *Listeria monocytogenes* prevalence in irrigation ponds by Mann-Whitney U test. (**A)** unidentified genus of order Actinomycetales (*p* = 0.022); **(B)** unidentified genus of family *Comamonadaceae* (*p* = 0.026); **(C)** *Rhizobacter* (*p* = 0.019); **(D)** *Janthinobacterium* (*p* = 0.029); **(E)** *Lysobacter* (*p* = 0.004); **(F)** *Blastomonas* (*p* = 0.01); and **(G)** unidentified genus of order Bacillales (*p* = 0.011). Lm denotes *Listeria monocytogenes.*

Based on the taxonomic database, correlation analysis and a Mann-Whitney test were conducted to identify potential indicators for *S. enterica* and *L. monocytogenes* contamination in irrigation water (Tables 5 and 6; Figures 5, 6). Fourteen bacterial genera and one genus were identified to be positively and negatively, respectively, correlated with *S. enterica* prevalence (Table 5). Abundance of an unidentified genus of the family *Sphingomonadaceae* and the genus *Hymenobacter* were associated with *S. enterica* presence (significantly correlated to population density or prevalence). Six bacterial genera under Sphingomonadales were significantly correlated with *S. enterica* presence in tested irrigation ponds (Table 5). In addition, the relative abundance of Sphingomonadales was positively correlated with *S. enterica* contamination in irrigation ponds (prevalence: correlation coefficient: 0.411485, *p*: 0.00498199; population density: correlation coefficient: 0.521871, *p*: 0.00023638). The relative abundances of three genera in *Sphingomonadaceae*, Sphingomonadales, and *Erythrobacteraceae* were significantly higher in *S. enterica* positive well water samples, while levels of *Fluviicola* were significantly higher in *S. enterica* negative samples (Figure 5).

**Table 5:** Bacterial genera (OTUs) significantly correlated with *Salmonella enterica* population density or prevalence in irrigation ponds on ESV.

Correlated bacterial genera	Pearson’s correlation coefficients to *Salmonella enterica* population density [log(MPN +1)]	*p*^a^	Biserial correlation coefficients to *Salmonella enterica* prevalence	*p*^b^
**Positive correlation (14)**				
**Unidentified genus of family *Sphingomonadaceae*** ^*^	0.33762	0.02333	0.34553	0.02009
*Massilia*	0.65318	1.15E-06	0.23088	0.12705
Sphingomonas^*^	0.42304	0.00379	0.288	0.05506
Sphingobium^*^	0.53573	0.00015	0.19148	0.20768
Unidentified genus of order Sphingomonadales^*^	0.19304	0.20393	0.39733	0.00688
*Albidiferax*	0.31742	0.03362	0.20241	0.18238
*Arthrobacter*	0.6389	2.32E-06	0.21472	0.15665
Unidentified genus of family Erythrobacteraceae^*^	0.16355	0.28308	0.31016	0.03813
***Hymenobacter*** ^*^	0.33546	0.02428	0.32893	0.02737
*Actinomycetospora*	0.26588	0.07752	0.31714	0.03378
*Aeromicrobium*	0.53457	0.00016	0.28236	0.06022
*Azospirillum*	0.65787	9.10E-07	0.24851	0.09976
Unidentified genus of family *Intrasporangiaceae*	0.66274	7.10E-07	0.23321	0.12314
*Terrabacter*	0.63048	3.45E-06	0.2034	0.18021
**Negative correlation (1)**				
*Fluviicola*	−0.2518	0.0952	−0.3119	0.03706

*Bacterial genera significantly correlated to both Salmonella enterica prevalence and population density are in bold*.

a Value of *p* of Biserial correlation coefficients.

b Value of *p* of Pearson’s correlation coefficients.

* Genera of order Sphingomonadales.

**Table 6:** Bacterial genera (OTUs) significantly correlated with *Listeria monocytogenes* population density or prevalence in irrigation ponds on ESV.

Correlated bacterial genera	Pearson’s correlation coefficients to *Listeria monocytogenes* population density [log(MPN +1)]	*p*^a^	Biserial correlation coefficients to *Listeria monocytogenes* prevalence	*p*^b^
**Positive correlation (17)**				
Unidentified genus of order Actinomycetales	0.22673	0.13422	0.36147	0.0147
**Unidentified genus of family *Comamonadaceae***	0.44855	0.002	0.33184	0.02596
Unidentified genus of family *Cytophagaceae*	0.21736	0.15151	0.3243	0.02975
Unidentified genus of family *Cryomorphaceae*	−0.0093	0.95165	0.30592	0.04098
*Rhodobacter*	0.29894	0.04607	0.07472	0.62568
Unidentified genus of family *Oxalobacteraceae*	0.17013	0.26389	0.2985	0.0464
*Dechloromonas*	0.30048	0.04491	0.07457	0.62638
*Duganella*	0.35229	0.01763	0.23624	0.1182
*Sphaerotilus*	0.33639	0.02386	0.28097	0.06155
*Steroidobacter*	0.11674	0.44505	0.33585	0.0241
*Albidiferax*	0.62127	5.26E-06	0.20742	0.17159
*Pseudonocardia*	0.3769	0.01071	0.16942	0.26589
Unidentified genus of order Chromatiales	0.14719	0.33461	0.34928	0.01869
*Sulfuricurvum*	0.17689	0.24509	0.33468	0.02463
***Rhizobacter***	0.40636	0.00561	0.33248	0.02565
*Janthinobacterium*	0.40279	0.00609	0.24654	0.50285
Unidentified genus of order Actinomycetales	0.22673	0.13422	0.36147	0.0147
**Negative correlation (3)**				
*Lysobacter*	−0.2116	0.1629	−0.3385	0.02298
*Blastomonas*	−0.2565	0.08911	−0.3125	0.03668
Unidentified genus of order Bacillales	−0.2901	0.05331	−0.3671	0.01314

Bacterial genera significantly correlated to both *Listeria monocytogenes* prevalence and population density are in bold.

a Value of *p* of Biserial correlation coefficients.

b Value of *p* of Pearson’s correlation coefficients.

Seventeen and three bacterial genera were identified to be positively and negatively correlated with *L. monocytogenes* prevalence, respectively (Table 5). Abundance of an unidentified genus of the family *Comamonadaceae*, and the genus *Rhizobacter* was associated with presence of *L. monocytogenes*. The relative abundance of the bacterial order Burkholderiales was significantly correlated with *L. monocytogenes* population density in irrigation ponds (correlation coefficient: 0.3138, *p*: 0.03580419). Abundance of four bacterial genera, including an unidentified genus of order Actinomycetales, one genus in the family *Comamonadaceae*, and the genera *Rhizobacter* and *Janthinobacterium* were significantly higher in *L. monocytogenes* positive pond water samples (Figures 6A–D). Three genera, *Lysobacter*, *Blastomonas*, and one unidentified genus of the order Bacillales, were negatively correlated with *L. monocytogenes* prevalence and had significantly lower abundance in non-contaminated pond samples (Figures 6E–G).

## Discussion

In previous study, the prevalence and dynamics of *S. enterica* in irrigation ponds and wells on the ESV was reported. Spatial (farm location) and temporal (weekly) differences for *S. enterica* occurrence in surface pond water was detected (Gu et al., 2019a). In this study, the prevalence and dynamics of *L. monocytogenes* in irrigation ponds and wells on the ESV was investigated. Antimicrobial susceptibility test was further performed on all the *S. enterica* isolates from previous study. Overall, all the ponds on the four studied farms tested positive for both foodborne pathogens at certain sampling points. *S. enterica* was isolated from three of the four wells, while *L. monocytogenes* was only detected from one sampled well during the study period. Antimicrobial resistant *S. enterica* was only identified from pond water samples, which may indicate a higher food safety risk of applying surface water for agricultural irrigation in this region. The average population densities of *S. enterica* and *L. monocytogenes* in tested water samples were lower than 2 log and 1 log MPN/L, respectively, but irrigation with naturally contaminated water might result in transmission of foodborne pathogens to fields (Gu et al., 2018). The results provide growers with baseline information on the risk of irrigation water being contaminated in this region. In this study, the correlation between *S. enterica* spp. and *L. monocytogenes* was further analyzed. *Salmonella enterica* MPN data from the selected weeks in which *Listeria* detection were conducted and used to compare the relationship of the two foodborne pathogens. In addition, the average *S. enterica* MPN/L of each month was calculated for comparison. However, neither the population of *S. enterica* per week nor average *S. enterica* values by month were correlated with *L. monocytogenes* prevalence in either pond or well water samples. Even though *L. monocytogenes* was detected in irrigation ponds and one well on the ESV, no listeriosis outbreaks and recalls have been traced back to this region. This might be explained by the low population of *L. monocytogenes* in irrigation water. Another hypothesis is that the agricultural and processing practices of food commodities produced in the tested area mitigate the survival or transmission rate of *L. monocytogenes*.

Correlation between foodborne pathogens (*S. enterica* spp. and *L. monocytogenes*) and contextual factors, including water parameters, weather information, and fecal indicators, was analyzed in this study. In contrast to a former study conducted in Georgia (Luo et al., 2016), no significant correlation between temperature and *S. enterica* prevalence was found in the tested irrigation ponds on the ESV. The low correlation coefficients (less than 0.15) between tested water parameters and foodborne pathogens in irrigation water indicate the weak potential of the tested physicochemical water characteristics as predictors in this region.

In agreement with multiple previous studies, fecal indicators were not significantly correlated with *S. enterica* and *L. monocytogenes* prevalence in sampled irrigation ponds (Ahmed et al., 2009; Benjamin et al., 2013; McEgan et al., 2013). The higher microbial populations in surface water and exposed environments with complex contamination routes might affect the sensitivity of using current fecal indicators for the prediction of foodborne pathogens in irrigation ponds. In addition, prevalence variance of different foodborne pathogens (*S. enterica* spp. and *L. monocytogenes*) in the same water samples as well as the high variance among different farms (location) bring challenges in identifying one universal indicator or standard to evaluate contamination risks in pond (surface) water. The low correlation coefficient between fecal indicators, including generic *E. coli*, and foodborne pathogens in irrigation ponds suggests further efforts to identify more suitable indicator microorganisms or other biological markers.

Microbial community analysis through 16S rDNA sequencing provides clues to identify suitable alternative indicators and potential suppressors of *S. enterica* and *L. monocytogenes* in pond irrigation water. For example, one unidentified genus of *Sphingomonadaceae* is positively correlated with *S. enterica* prevalence and population. *Sphingomonadaceae* is a family of class Alphaproteobacteria. It is known by the ability of some species to degrade aromatic compounds, which is important to environmental remediation and may benefit the survival of *S. enterica* spp. in pond water. Another five genera of order Sphingomonadales were also determined to be positively correlated with *S. enterica* prevalence or population (Table 5). The interaction between associated bacterial genera of order Sphingomonadales and *S. enterica* contributed to the positive correlation in irrigation ponds is unclear. Further studies to investigate the microbial relationship may benefit the identification of specific factors that affect *S. enterica* contamination in surface water. The genus *Rhizobacter* was positively correlated with *L. monocytogenes* prevalence and population. *Rhizobacter* includes species isolated from the plant rhizosphere. The significant positive relationship between these bacteria and *L. monocytogenes* indicates that the contamination of this foodborne pathogen may be associated with run off from plant rhizosphere soil into irrigation ponds. In addition, the positive correlation between water turbidity and *L. monocytogenes* presence supports the hypothesis of contamination through external sources, like soil. Bacillales is an order of Gram-positive bacteria, which includes the *Listeria* spp. One unidentified genus of this order is negatively correlated with the foodborne pathogen, which may be caused by its competitive relationship to *L. monocytogenes* in the environment. Species of Sphingomonadales and *Rhizobacter* should be further examined as alternative indicator microorganisms for *S. enterica* spp. and *L. monocytogenes* in irrigation ponds, respectively. The diverse and complex composition of microbial communities in irrigation water are associated with environmental conditions and water sources (Gu et al., 2019b; Chung et al., 2020). The sample size (*n* = 96) performed for high-throughput sequencing in this study might be limited to cover the comprehensive microbial communities. Further research with larger sample size and higher sequencing depth for microbiome study on irrigation water is suggested to evaluate identified bacterial taxa as indicators or biocontrol agents of foodborne pathogens.

The population density of fecal indicators, coliform and generic *E. coli*, are statistically correlated with *S. enterica* prevalence in irrigation wells. However, correlation coefficients between fecal indicators and *S. enterica* were less than 0.3 (Table 2). If we analyze the correlation to each individual well, coliform and generic *E. coli* were only significantly correlated with *S. enterica* in well D but not the other wells. In addition, they are not correlated with *L. monocytogenes* in well water. Thus, fecal indicators may not be good enough as a general standard to assess the contamination of foodborne pathogens in tested irrigation sources. Due to the different spatial distribution and occurrence of *S. enterica* and *L. monocytogenes*, it would not be beyond our expectations that different bacterial taxa were correlated with these two foodborne pathogens in sampled irrigation water. It is notable that pond D had the highest *S. enterica* MPN values, and the prevalence of *S. enterica* was considerably higher in well D compared to ponds and wells in other farms. The relative high levels of *S. enterica* in this farm might be associated with distinct contamination sources compared to other farms on ESV, which results in significant correlation to water turbidity in ponds and fecal indicator population in well water samples. Nevertheless, even though farm D was observed to have relative higher risk of *S. enterica* contamination in pond and well water, the levels of fecal indicators, coliform and generic *E. coli*, were not the highest among all tested farms (Figure 2).

In addition to environmental factors mentioned above, fertilization with poultry litter in this agricultural region might be associated with the contamination of *S. enterica* in irrigation water (Gu et al., 2019a). *S. enterica* contamination of fresh produce is often linked to animal farming operations (Volkova et al., 2009; Rothrock et al., 2017), nevertheless there are evidences that many serovars have increased fitness for survival and growth in the natural environments independent of animal hosts (Brandl and Mandrell, 2002; Brandl et al., 2006, 2013). *S. enterica* serovar Newport was implicated in the recurring outbreaks associated with tomato in this agricultural region (Greene et al., 2008; Bennett et al., 2015). Diverse *S. enterica* serotypes were identified from irrigation water, poultry litter, and field soil samples in this region (Gu et al., 2019a,b). However, serovar Newport was identified to be the dominant serotype in sampled irrigation water (35% in tested pond water and 65% in well water), but not in poultry litter (2%) or poultry litter amended field soil (6%) samples. This is in agreement with another survey study about the prevalence of *S. enterica* in environmental samples in this agricultural region (Bell et al., 2015), which indicated irrigation with contaminated water might be the main source of the specific *S. enterica* serotype identified from multiple salmonellosis outbreaks and traced back to this region. This may also be associated with the survival advantages of the specific *S. enterica* Newport strain in irrigation water and on tomato. It is hard to demonstrate the direct link between poultry litter application and contamination of *S. enterica* in irrigation water and on produce in this area since farms tested for different types of samples were geographically distinct (distance > 3 km).

Those results are based on a 1-year study with limited sample size (*n* = 96) for *L. monocytogenes* test and microbiome analysis, thus there are limits to the conclusions that can be drawn. The work presented here, demonstrates current fecal indicators may not be adequate for the evaluation of agricultural water for produce safety, especially for surface water sources. Different from several published studies (Haley et al., 2009; Walters et al., 2011; Harris et al., 2018), significant correlations between weather parameters, including rainfall and temperature, were not evidenced in this research. The “negative” correlation is also important to be reported because it provides data that on-farm contamination events might be driven by geographical factors. Therefore, data might be needed in different agricultural regions to describe how weather could influence pathogen dynamics. 16S rDNA sequencing provides clues to identify alternative indicator and suppressors of foodborne pathogens in irrigation water. Further metagenomics analysis, such as shotgun sequencing to classify the microbial communities and functional analysis, can shed light to screen and identify practical indicator microorganisms or other biological markers that could reliably indicate the presence or absence of pathogenic viruses, protozoa, parasites, and bacteria in agricultural water.

## Data Availability Statement

The original contributions presented in the study are included in the article/Supplementary Material, further inquiries can be directed to the corresponding author.

## Author Contributions

GG and SR contributed to the study conception and design. GG, AO, PR, ER, JZ, and RB contributed to the acquisition of data. GG and LS contributed to analysis and interpretation of data. GG contributed to drafting of manuscript. LS, AO, ER, and JZ contributed to critical revision. All authors contributed to the article and approved the submitted version.

### Conflict of Interest

The authors declare that the research was conducted in the absence of any commercial or financial relationships that could be construed as a potential conflict of interest.
