# Supplementary material for: Correlation of Salmonella enterica and Listeria monocytogenes in Irrigation Water to Environmental Factors, Fecal Indicators, and Bacterial Communities
Source: Front Microbiol. 2021 Jan 8;11:557289. doi: 10.3389/fmicb.2020.557289 (PMC7820387; doi:10.3389/fmicb.2020.557289)
Supplement: Supplementary file 1 [file Data_Sheet_1.pdf]

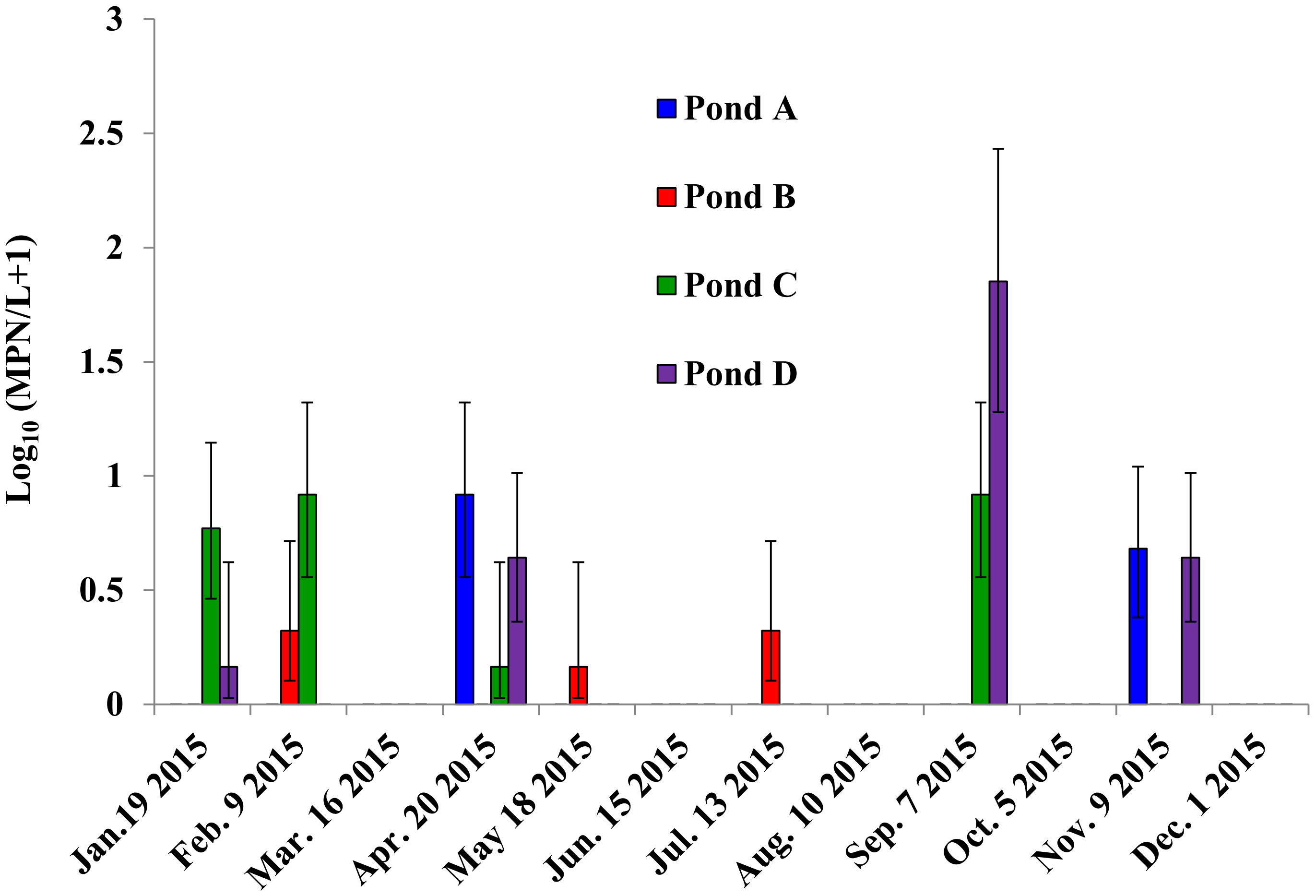

Figure S1. *Salmonella* MPN values of selected monthly samples (for both foodborne pathogen testing and sequencing) in the irrigation ponds of tested four farms A-D on the Eastern Shore of Virginia (ESV). Bars represent 95% confidence intervals.

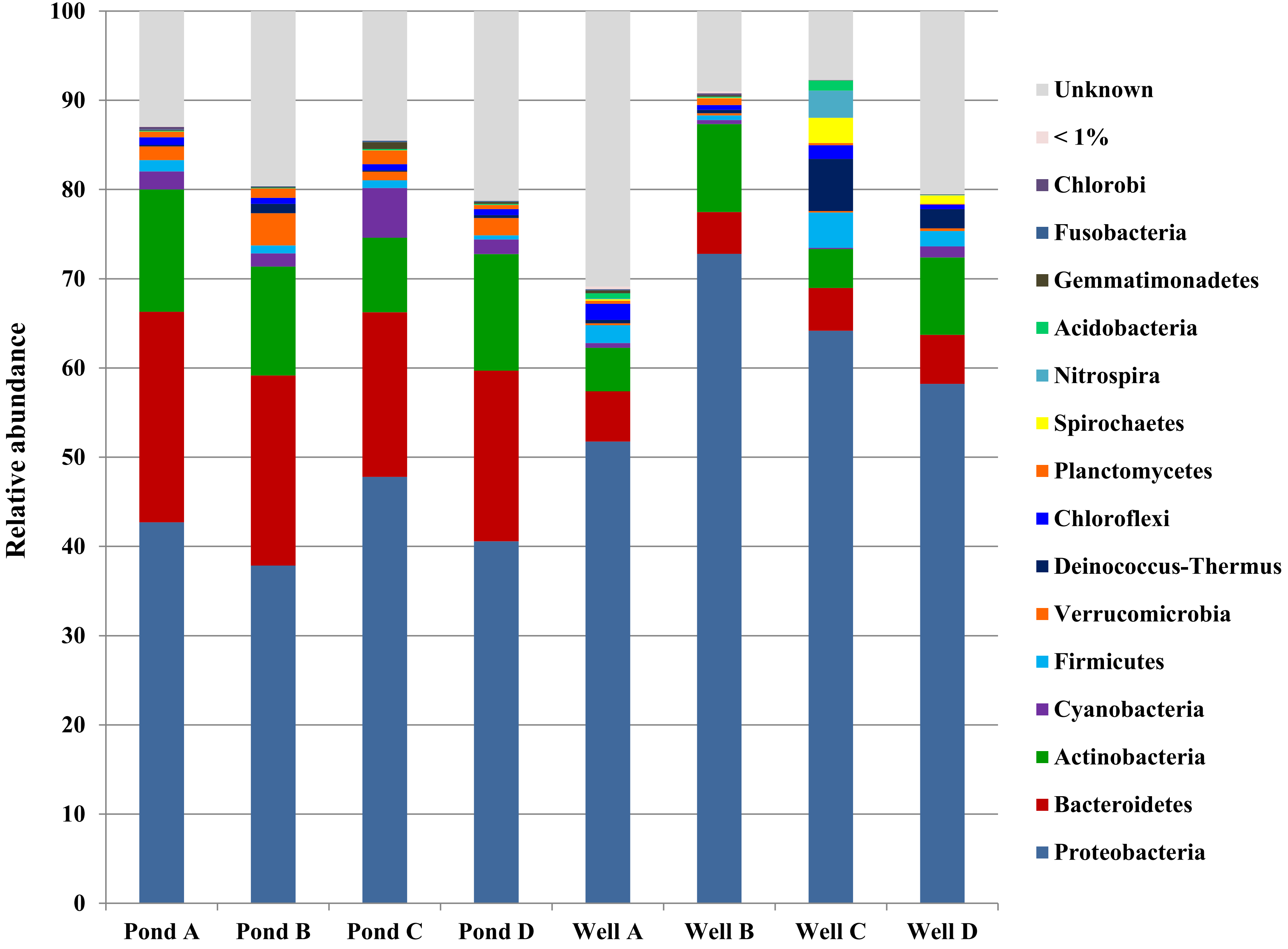

Figure S2. Phylum-level classification of the sequence reads of samples from the eight tested water sources. <1%, minor orders with abundance of <1%.

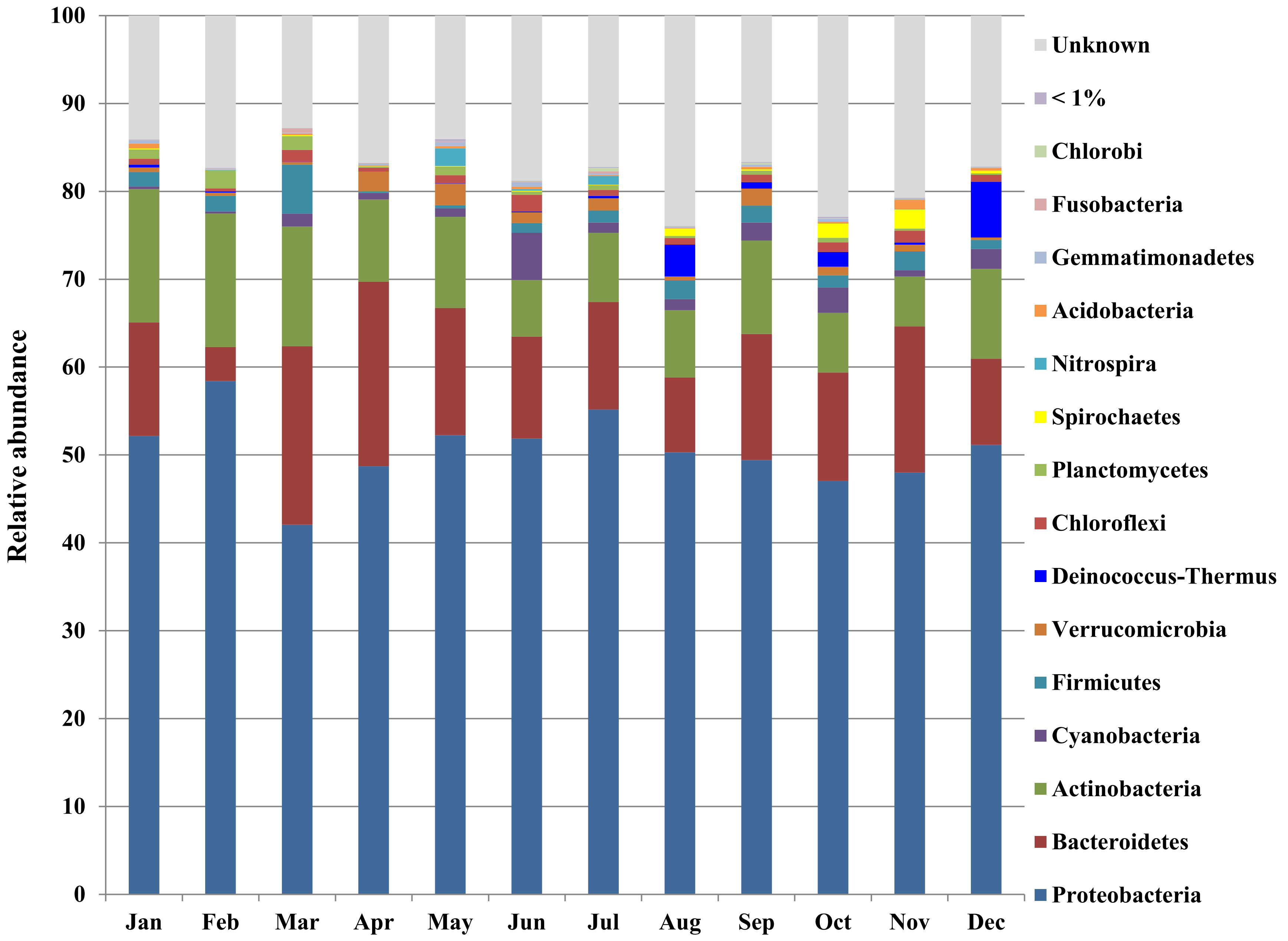

Figure S3. Dynamics of bacterial taxa (phylum) among sampling months.

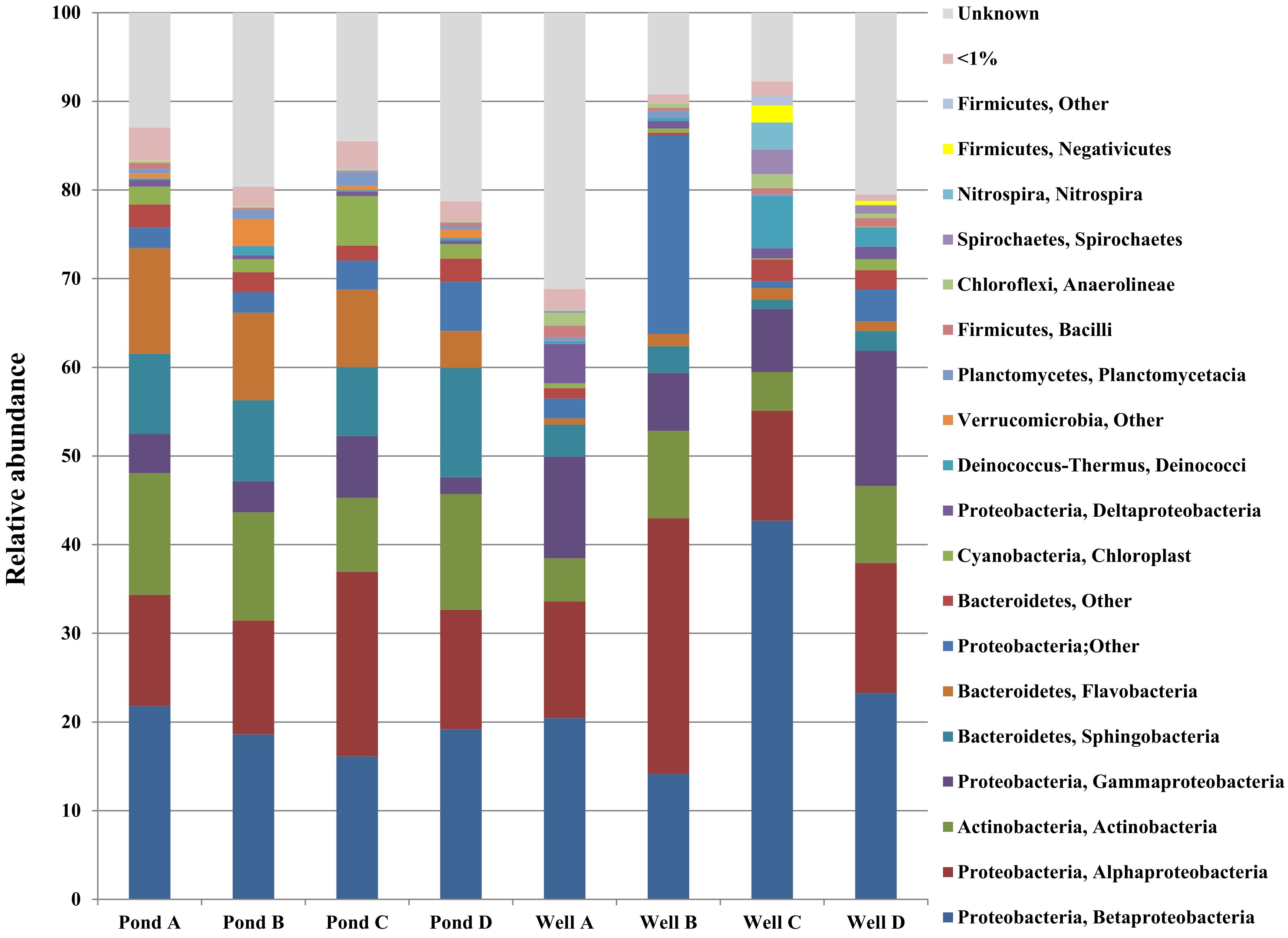

Figure S4. Class-level classification of the sequence reads of samples from the eight tested water sources. <1%, minor orders with abundance of <1%.

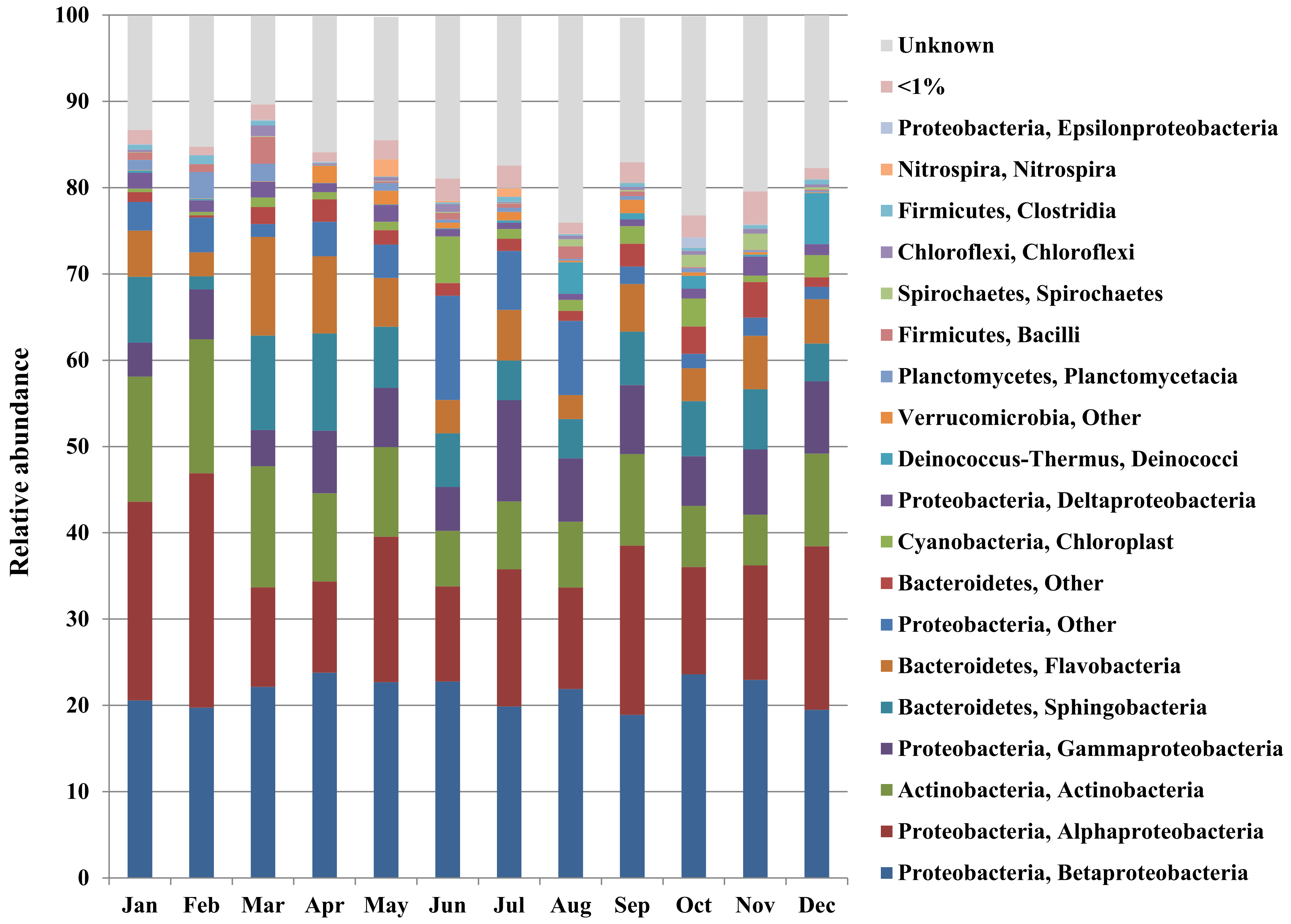

Figure S5. Dynamics of bacterial taxa (class) among sampling months.
