# Supplementary material for: Correlation of Salmonella enterica and Listeria monocytogenes in Irrigation Water to Environmental Factors, Fecal Indicators, and Bacterial Communities
Source: Front Microbiol. 2021 Jan 8;11:557289. doi: 10.3389/fmicb.2020.557289 (PMC7820387; doi:10.3389/fmicb.2020.557289)
Supplement: Supplementary file 2 [file Data_Sheet_2.docx]

Table S1. Relative abundance (mean ± standard error) of bacterial phylum in sampled irrigation ponds and wells.

| Bacteria Phylum | p-mean | w-m |
| --- | --- | --- |
| **Proteobacteria** | 42.290 ± 1.538 | 60.404 ± 1.851 |
| **Bacteroidetes** | 20.631 ± 1.398 | 5.222 ± 0.821 |
| **Actinobacteria** | 11.732 ± 0.729 | 6.778 ± 0.915 |
| **Cyanobacteria** | 2.738 ± 0.665 | 0.629 ± 0.249 |
| **Firmicutes** | 0.884 ± 0.177 | 2.068 ± 0.408 |
| **Verrucomicrobia** | 2.021 ± 0.368 | 0.230 ± 0.042 |
| Deinococcus-Thermus | 0.423 ± 0.251 | 2.082 ± 0.766 |
| Chloroflexi | 0.739± 0.110 | 1.151 ± 0.188 |
| Planctomycetes | 0.929 ± 0.201 | 0.333 ± 0.091 |
| **Spirochaetes** | 0.026 ± 0.004 | 0.939 ± 0.391 |
| Nitrospira | 0.013 ± 0.003 | 0.725 ± 0.402 |
| **Acidobacteria** | 0.087 ± 0.015 | 0.462 ± 0.128 |
| Gemmatimonadetes | 0.299 ± 0.083 | 0.151 ± 0.035 |
| Fusobacteria | 0.106 ± 0.37 | 0.040 ± 0.024 |
| Chlorobi | 0.071 ± 0.056 | 0.052 ± 0.034 |
| < 1% * | 0.057 ± 0.007 | 0.153 ± 0.028 |
| Unknown | 16.956 ± 1.687 | 18.583 ± 1.964 |

Phylum in bold have significant relative abundance between pond and well water; * phylum with relative abundance less than 1 %.

Table S2. Relative abundance (mean ± standard error) of bacterial classes in sampled irrigation ponds and wells.

| Phylum, Class | Pond-mean | well-mean |
| --- | --- | --- |
| **Proteobacteria, Betaproteobacteria** | 18.840 ± 0.783 | 24.953 ± 1.896 |
| Proteobacteria, Alphaproteobacteria | 15.051 ± 1.255 | 16.460 ± 1.503 |
| **Actinobacteria, Actinobacteria** | 11.732 ± 0.729 | 6.778 ± 0.915 |
| **Proteobacteria, Gammaproteobacteria** | 4.288 ± 0.614 | 10.594 ± 1.132 |
| **Bacteroidetes, Sphingobacteria** | 9.466 ± 0.718 | 2.531 ± 0.540 |
| **Bacteroidetes, Flavobacteria** | 8.804 ± 1.020 | 1.089 ± 0.280 |
| Proteobacteria;Other | 3.304 ± 0.427 | 6.212 ± 1.955 |
| Bacteroidetes, Other | 2.253 ± 0.229 | 1.558 ± 0.343 |
| Cyanobacteria, Chloroplast | 2.738 ± 0.665 | 0.629 ± 0.249 |
| **Proteobacteria, Deltaproteobacteria** | 0.529 ± 0.065 | 2.168 ± 0.329 |
| Deinococcus-Thermus, Deinococci | 0.423 ± 0.251 | 2.082 ± 0.766 |
| **Verrucomicrobia, Other** | 1.291 ± 0.340 | 0.045 ± 0.015 |
| **Planctomycetes, Planctomycetacia** | 0.879 ± 0.202 | 0.318 ± 0.091 |
| Firmicutes, Bacilli | 0.360 ± 0.090 | 0.893 ± 0.255 |
| **Chloroflexi, Anaerolineae** | 0.096 ± 0.018 | 1.006 ± 0.186 |
| **Spirochaetes, Spirochaetes** | 0.026 ± 0.004 | 0.939 ± 0.391 |
| Nitrospira, Nitrospira | 0.013 ± 0.003 | 0.725 ± 0.402 |
| **Firmicutes, Negativicutes** | 0.030 ± 0.038 | 0.571 ± 0.140 |
| Firmicutes, Other | 0.063 ± 0.286 | 0.2575 ± 0.280 |
| <1% | 2.838 ± 1.688 | 2.033 ± 1.978 |
| Unknown | 17.005± 0.005 | 18.732 ± 0.222 |

Classes in bold have significant relative abundance between pond and well water; * classes with relative abundance less than 1 %.
